# Characteristics of the Enterococcus Phage vB_EfS_SE, and the Properties of Its Chimeric Endolysins Harboring a PlySE-Carbohydrate-Binding Domain and a Synthetic Enzymatic Domain

**DOI:** 10.3390/pharmaceutics16101312

**Published:** 2024-10-09

**Authors:** Rustam M. Buzikov, Vladislav A. Kulyabin, Olga N. Koposova, Vyacheslav A. Arlyapov, Andrey M. Shadrin

**Affiliations:** 1 Laboratory of Bacteriophage Biology, G.K. Skryabin Institute of Biochemistry and Physiology of Microorganisms, Pushchino Scientific Center for Biological Research of the Russian Academy of Sciences, Federal Research Center, Prospect Nauki, 5, 142290 Pushchino, Russia; brust@pbcras.ru (R.M.B.); kuliabin.vlad@pbcras.ru (V.A.K.); koposova@pbcras.ru (O.N.K.); 2 Research Center “BioChemTech”, Tula State University, 300012 Tula, Russia; v.a.arlyapov@tsu.tula.ru

**Keywords:** bacteriophage, endolysin, antibiotic resistance, phage therapy, *Saphexavirus*, *Enterococcus faecalis*, *Enterococcus faecium*

## Abstract

**Background/Objectives:** The World Health Organization has selected enterococci as one of the priority multidrug-resistant microorganisms for the development of new antibacterial drugs. Bacteriophages are promising antibacterial agents, but the biology of bacteriophages requires deeper understanding. **Methods:** The vB_EfS_SE phage which is capable of infecting four species of the genus *Enterococci* was isolated from sewage plant. The complete genome of the vB_EfS_SE phage was sequenced using illumina technology. The endolysin gene was cloned into pBAD18 expression vector. Two chimeric endolysins were engineered using the vB_EfS_SE carbohydrate-binding domain (CBD) and replacing its enzymatically active domain (EAD). **Results:** The bacteriophage exhibits promising lytic properties and persists at temperatures of 40 °C and below, and under pH conditions ranging from 5 to 11. The genome sequence is 57,904 bp in length. The vB_EfS_SE endolysin PlySE and chimeric endolysins PlyIME-SE and PlySheep-SE were found to have the same range of specificity, but different thermostability properties and a different pH range for enzyme activity. **Conclusions:** Taking together the results obtained in this work and other published studies, we can highly appreciate the potential of *Saphexavirus* phages and their endolysins as novel antibacterial compounds.

## 1. Introduction

Enterococci are normal human microbiota. Enterococcal cell concentrations can reach more than 10^8^ per gram of feces in infants, but are usually much lower in adults [1]. Certain enterococci strains are capable of causing dangerous bacterial infections of the urogenital tract, wound fever, neonatal sepsis, meningitis, bacteremia, and endocarditis [2,3,4]. Pathogenic properties of strains are determined by the presence of pathogenicity factor genes or certain pathogenic alleles of genes.

The number of deaths caused by antibiotic-resistant bacteria is growing every year. In 2019, antibiotic-resistant *E. faecalis* and *E. faecium* were among the leading bacterial species causing fatal bacterial infections [5]. According to WHO Bacterial Priority Pathogens List, 2024 update, vancomycin-resistant *E. faecium* is in the High priority group for developing novel antibacterial compounds [6].

Bacteriophages and phage-derived enzymes are a growing category of new antibacterial drugs. According to the 2023 WHO overview, there are 17 bacteriophage-based drugs at different stages of clinical trials [7]. In some countries, such as Georgia and Russia, bacteriophages are used in therapy. In the Russian Federation, testing of microflora for sensitivity to therapeutic phage preparations is available in network diagnostic centers throughout the country.

Another promising antibacterial agent source is phage-derived enzymes. A number of phage lytic enzymes that act from the inside of the cell are produced in the end of the lytic cycle: holins, which form pores in the bacterial membrane, and endolysins, which degrade the peptidoglycan layer of the cell wall. Together these molecules act to release phage progeny from the infected cell. Since gram-positive bacteria lack an outer membrane, their peptidoglycan layer can be degraded by endolysins acting from the outside of the cell [8].

In this work we describe the physiological properties of the bacteriophage vB_EfS_SE and determine its complete genome sequence. In addition, we compare the properties of the PlySE endolysin with two chimeric endolysins: PlyIME-SE and PlySheep-SE, consisting of the PlySE carbohydrate-binding domain and homologues of the PlySE catalytic domain.

## 2. Materials and Methods

### 2.1. Bacterial Strains

The *E. faecium*, *E. faecalis*, *E. thailandicus*, *E. hirae*, *E. avium*, *E. durans*, and *E.* sp. strains were obtained from All-Russian Collection of Microorganisms (VKM) and Biological Resource Center Russian National Collection of Industrial Microorganisms (VKPM) collections. The cultures were grown at 35 °C in LB medium (per liter of medium: 10 g tryptone, 5 g yeast extract, and 10 g NaCl), supplementing the medium with 0.5% or 1.5% agar if necessary. The culture growth was assessed by changes in the optical density of the culture at 595 nm on a FilterMax F5 plate reader (Molecular Devices, San Jose, CA, USA).

### 2.2. Phage Isolation

Bacteriophage vB_EfS_SE was isolated from the Pushchino (Moscow Oblast, Russia) wastewater treatment plant as previously described for the Izhevsk phage [9], and aliquots were plated on Petri dishes using the two-layer agar method on the lawns of the VKPM B-12629 strain. Samples of the top agar layer containing a single negative colony were transferred to Eppendorf tubes with 1 mL of SM+ (10 mM NaCl, 1 mM MgSO_4_, 50 mM Tris-HCl pH 7.5, 0.01% gelatin) and 100 μL of chloroform. Extraction of virions from the samples was performed by shaking for four hours on a Bio RS-24e rotator (Biosan, Riga, Latvia) at 25 °C. The tubes were then left to stand for 5 min to allow the chloroform to settle. A total of 50 μL of the extract was transferred to a plate containing 5 μL of the overnight culture of VKPM B-12629 strain and 500 μL of LB medium. The cultures were grown in a shaker overnight at 35 °C and 180 rpm. A series of tenfold dilutions was prepared from the lysed cultures in SM+ buffer and plated on double-layer LB agar to obtain single negative colonies of the phage. Such extraction-enrichment cycles were repeated until three consecutive passages no longer revealed differences in plaque morphology. The resulting lysate was used to produce purified phage in preparative quantities.

### 2.3. Production of Preparative Quantities of Phage, Purification, Titer Determination and Storage

A total of 2 mL of an overnight culture of *E. faecalis* VKPM B-12629 and 10, 100, or 200 μL of the phage lysate were added to shaking flasks with 50 mL of LB. Then, 100 μL of SM+ buffer was added to the control flask instead of the phage. Cultivation was carried out in a shaker-incubator (180 rpm, 35 °C) until cell lysis occurred. Following this, 2.9 g of sodium chloride was dissolved in 50 mL of the lysate, 0.5 mL of chloroform was added, and the mixture was shaken for 30 min. The lysate was centrifuged for 10 min at 11,500× *g* to remove bacterial debris. The supernatant was poured into a shaking flask, and 5 g of PEG-8000 was added to each 50 mL of the supernatant. After PEG-8000 was dissolved, the mixture was incubated for an hour at 6 °C. The suspension was centrifuged at 11,500× *g* for 10 min. The precipitated phage virions were resuspended in 5 mL of SM buffer. The suspension was filtered through a 0.2 μm PES filter.

The last stage of purification was centrifugation in a CsCl-step density gradient (1.45 g/mL, 1.5 g/mL, and 1.7 g/mL) for 3 h at 178,038× *g* in a Beckman L7 ultracentrifuge, followed by dialysis against SM buffer.

The concentration of phage was determined by tenfold dilutions on a two-layer agar with *E. faecalis* VKPM B-12629. Long-term storage of the phage was carried out at −20 °C in the presence of 15% glycerol.

### 2.4. Determination of Phage Host Range

The bacteriophage host range was evaluated using spot test and liquid culture lysis techniques. If one of the methods showed bacterial lysis, the culture was considered sensitive. For spot tests, 5 μL of SM+ buffer containing phage vB_EfS_SE at a concentration of 10^2^ to 10^10^ PFU/mL was applied to plates inoculated with overnight cultures of different enterococci strains from the laboratory collection. The minimum required amount of inoculum for lawn formation was selected for each strain individually. The experiment was repeated three times. The results were evaluated after 24 h of incubation at 35 °C. For liquid culture lysis, the cultivation was carried out in 48-well plates in a FilterMax F5 reader. A total of 400 μL of LB medium containing bacterial cells in the exponential growth phase (OD595 = 0.4) was mixed with the phage at MOI = 1. Sterile SM+ buffer was added to the control wells. Cultivation was carried out at 300 rpm and 35 °C overnight. The experiment was repeated three times. In cases where the growth curve of a tested sample was statistically significantly different from that of the control, the shape of the curve was analyzed, and conclusions were made about the nature of phage–cell interaction (pronounced lysis of the culture was defined as productive interaction, and growth inhibition/bacteriostatic effect as unproductive interaction).

### 2.5. Phage DNA Extraction, Purification, Sequencing and Genome Assembly

The phage preparation obtained as described in Section 2.3 was treated with RNaseA (EN0531, Thermo Scientific Baltics UAB, Vilnius, Lithuania) and DNAseI (NEB, #M0303S, Ipswich, MA, USA) at 37 °C for an hour with occasional stirring. Then, the capsids were lysed by SDS and proteinase K [10] and the DNA was purified with phenol-chloroform extraction. The quality and concentration of the resulting phage DNA preparation were assessed using agarose gel electrophoresis in 0.7% agarose and spectrophotometry using NanoPhotometer Pearl P-360 (Implen GmbH, Munich, Germany). The paired-end Illumina sequencing library was constructed using KAPA HyperPlus Kits (Kapa Biosystems, Wilmington, MA, USA) and was sequenced on the Illumina MiSeq platform. Raw reads were subsampled to 20% using the Sub-sample sequences files tool [11], trimmed and filtered for >90% quality with Trimmomatic [12], and then quality-controlled with FastQC version 0.11.7 [13]. The genome assembly was performed in the SPAdes v.3.14.1 software [14]. For subsequent evaluation of the results, the Bandage program [15] was used, and the read coverage of the assembly was assessed in Tablet 1.21.02.08 [16].

### 2.6. Genome Annotation

Preliminary genome annotation was performed using the Pharokka v 1.2.0 utility [17]. Further verification was performed using HHpred Version: 57c8707149031cc9f8edceba362c71a3762bdbf8 and BLASTp V2.16.0, and based on the analysis of the obtained data (Supplementary Table S1), the genome was annotated and uploaded to GenBank (PP583004.1). The genome map was visualized in CGview V7.

### 2.7. Phylogenetic Analysis

A preliminary search for related genomes of the vB_EfS_SE phage was performed using NCBI BLASTn and BLASTx V2.16.0. Then, using the ViPtree software V4.1, a putative proteome of vB_EfS_SE was obtained based on the phage genome, and it was compared with other viruses. Enterococcus phage vB_OCPT_CCS3 (ON113174), Enterococcus phage vB_OCPT_CCS2 (ON113174), Enterococcus phage SSsP-1 (MZ333457), Enterococcus phage EF_RCK (PP028463), and Enterococcus phage EFKL (OP831581) genomes were added manually.

### 2.8. Transmission Electron Microscopy

Transmission electron microscopy was performed as described before using a JEM-100C (JEOL, Akishima, Japan) transmission electron microscope with 80 kV accelerating voltage, Kodak film SO-163 (Kodak, Cat.# 74144, Hatfield, PA, USA), and ImageJ version 1.53e [https://imagej.nih.gov/ij/index.html, accessed on 1 June 2021] software for determining the virion size [18].

### 2.9. Killing Assay and the Effect of Ca^2+^ and Mg^2+^ Salts

One milliliter of an overnight culture was added to a test tube with LB medium and grown to OD595 = 0.4, which corresponds to the exponential growth phase. After that, the culture was added to a 48-well plate (360 μL per well), and mixed with 40 μL of phage suspensions with different titers. CaCl_2_ and MgCl_2_ were added to the SM+ buffer to a final concentration of 10 mM. Buffer without phage was added as a control, and LB without cells with SM+ and the corresponding salts were added as blanks. The cultivation lasted 2.5–3.5 h, and obtained growth curves were subjected to statistical processing by the ANOVA method.

### 2.10. Bacteriophage Thermal and pH Stability

To determine thermal stability, phage aliquots were incubated at temperatures from 10 to 80 °C for an hour. After incubation, the retained concentration of viable virions was determined by the double-layer agar method. To determine the pH-stability range of the vB_EfS_SE bacteriophage, 10 μL of a bacteriophage preparation was mixed with 90 μL of a buffer solution (pH from 2.2 to 12.0) and incubated for one hour at 20 °C. After that, a residual phage concentration was determined using the double-layer agar method. The following set of buffer solutions was used to create conditions with specific pH values: 0.05 M glycine-HCl (pH 2.2, 3.0); 0.2 M sodium acetate (pH 4.0, 5.0); 0.1 M sodium phosphate (pH 6.0, 7.0, 8.0); and glycine-NaOH (pH 9.0, 10.0, 11.0 and 12.0). Both experiments were carried out in five replicates and subjected to statistical analysis using the ANOVA method.

### 2.11. Cloning of Endolysin Genes

All expression vectors were assembled using the TEDA (T5 exonuclease DNA assembly) method [19]. The *ply*SE gene was PCR amplified using high-fidelity Q5 DNA polymerase (NEB) in the presence of specific primers (Supplementary Table S2). The pBAD18 plasmid was treated with restriction endonucleases HindIII (NEB) and XbaI (NEB). The treated pBAD18 plasmid and the *ply*SE gene PCR product were added to 4 μL of 5× TEDA solution (0.5 M Tris–HCl pH 7.5; 50 mM MgCl_2_; 50 mM DTT; 0.25 g PEG 8000; and 0.01 U/μL T5 exonuclease) in a molar ratio of 1:3/1:4, and the final volume was brought to 20 μL. The mixture was incubated for 40 min at 30 °C, cooled on ice, and then transformed into competent *E. coli* XL1 Blue cells. The resulting pBAD18-plySE plasmid was used to obtain the PlySE protein containing the N-terminal amino acid sequence with six histidines for subsequent purification. To obtain the fusion proteins PlyIME-SE and PlySheep-SE, the pBAD18-PlySE vector was digested with NcoI (NEB) and HindIII (NEB) restriction endonucleases. The regions of genes containing the CHAP domains of PlyIME-EF1 [20], PlySheep (GenBank: EGO5065395.1), and the CBD domain of PlySE were amplified with Q5 DNA-polymerase (NEB) using primers with 5′-ends complementary to the plasmid (Supplementary Table S1). Plasmid assembly was carried out using the TEDA method with plasmid and two PCR products corresponding to the CHAP domain of endolysin of interest and CBD domain of PlySE. The linker region was part of the PlySE CBD domain PCR product (Supplementary Figure S2).

### 2.12. Expression and Purification of Endolysins

Plasmids were transformed into *E. coli* TOP10. An overnight culture of the recombinant strain was diluted (1:100) in 500 mL LB and grown to OD590 = 0.4–0.6 at 37 °C in an orbital shaker. Induction of target proteins was performed by adding arabinose to a concentration of 0.1% (*w*/*v*) at 25 °C, and subsequent incubation overnight (12–16 h). Cells were pelleted by centrifugation at 8000× *g*, 4 °C for 7 min. The pelleted cells were then resuspended in 30–40 mL buffer A (40 mM Tris-HCl pH 8.0; 0.5 M NaCl; 5% glycerol), placed on ice, and homogenized using a Q700 Sonicator (Qsonica, Newtown, CT, USA). Three rounds of sonication for 30 sec at 50% amplitude were performed, with 5 min cooling between rounds. The cell lysate was centrifuged at 11,500× *g*, 4 °C for 50 min. The supernatant was filtered through a 0.45 μm filter (Milipore, Bedford, UK). The filtered lysate was loaded into a 5 mL Ni-chelate column (Hi-Trap, GE Healthcare, Upsala, Swidden), washed with buffer A with 50 mM imidazole. Target proteins were eluted with buffer A containing 300 mM imidazole. The quality and molecular weight of proteins in purified fractions were assessed by 15% SDS-PAGE (Supplementary Figure S3). Then, the fraction with the target protein was dialyzed against buffer D (20 mM HEPES pH 7.5; 0.5 M NaCl; 5% glycerol) with two buffer changes after 4 h each. In the last step, the protein was dialyzed against storage buffer S (20 mM HEPES pH 7.5; 0.5 M NaCl; 50% glycerol; 1 mM DTT). Protein concentration was determined by measuring absorbance at 280 nm using a NanoPhotometer Pearl P-360 (Implen GmbH, Munich, Germany). Proteins were stored in 80 µL aliquots at −80 °C. For further experiments, proteins were thawed and kept on ice until use.

### 2.13. Bacteriolytic Spectrum of Endolysins

The specificity of endolysins was determined in a spot test in double-layer agar on 26 strains of *Enterococcus*, *E. coli* XL1Blue, and *B. cereus* VKM B-370. The studied endolysins were diluted in 10 mM Tris-HCl pH 7.5 buffer to a final concentration of 1 µM. Then, 2 µL of diluted endolysin was applied to an agar plate with a tested strain lawn. Diluted protein storage buffer (buffer S) was used as a control. All plates were incubated at 35 °C overnight and analyzed for the appearance of cleared zones in the lawns.

### 2.14. Turbidimetric Assay of Endolysin Activity

To determine the bacteriolytic activity of endolysins, *E. faecalis* VKPM B-12629 cell culture was grown to OD590 ~0.4 and harvested by centrifugation at 5000× *g* and 4 °C for 7 min. The pellet was then resuspended in buffered LB (10 g/L tryptone; 5 g/L yeast extract; 10 g/L NaCl; 0.2 g/L KCl; 1.44 g/L Na_2_HPO_4_; 0.24 g/L KH_2_PO_4_; pH 7.5). The final OD595 value was adjusted to 0.4. The studied endolysins were diluted in buffered LB to a concentration of 10 µM; diluted protein storage buffer S was used as a control. A total of 20 µL of diluted endolysins was added to 380 µL of bacterial suspension in a 48-well plate to a final concentration of 0.5 µM. Incubation was carried out for 3 h with constant shaking at 35 °C in a FilterMax F5 plate reader (Molecular Devices, San Jose, CA, USA) with OD595 measuring every 5 min. Each experiment was conducted in three independent biological replicates. The results were interpreted using the GraphPad Prism program.

### 2.15. pH Optimum and Thermal Stability of Endolysins

To determine the optimal pH range for the bacteriolytic activity of endolysins, the same buffer solutions were used as in the bacteriophage pH stability assay. Instead of an LB buffered solution, a set of buffers with different pH values in the range from 2.2 to 10 was used. Enzyme activity was measured by turbidimetry assay (Section 2.14) with 1 h incubation at 35 °C. To assess the thermal stability of endolysins, aliquots of diluted endolysins (20 µM) were incubated at different temperatures ranging from 10 to 70 °C for 1 h. After incubation, residual enzyme activity was determined using a turbidimetric method. The experiments to determine the pH optimum and thermal stability were repeated five times independently. Enzyme activity was expressed as a percentage, with 100% representing the maximum observed activity of endolysins in the experiment. Results were presented using a box plot with a confidence interval of 5–95%. GraphPad Prism 8.4.3 was used for data visualization.

## 3. Results

### 3.1. Isolation and Specificity Range

The bacteriophage vB_EfS_SE, which infects bacteria of the genus *Enterococcus*, was isolated from the wastewater treatment plant (Pushchino, Russia, Moscow Oblast). The vB_EfS_SE bacteriolytic spectrum was tested on 26 strains of enterococci from the laboratory collection (Table 1). The bacteriophage vB_EfS_SE was able to infect 4 of the 26 (15%) tested strains. The four sensitive strains belonged to four different species: *E. avium*, *E. faecalis*, *E. faecium*, and *E. hirae*.

### 3.2. Plaques and Virion Morphology

On the lawns of *E. avium* VKM B-1673, *E. faecalis* VKPM B-12629, *E. faecium* VKPM B-4054, and *E. hirae* VKPM B-12152, the phage formed transparent plaques with a smooth edge and a diameter of about 1–1.7 mm (Supplementary Figure S1).

**Figure 1:**
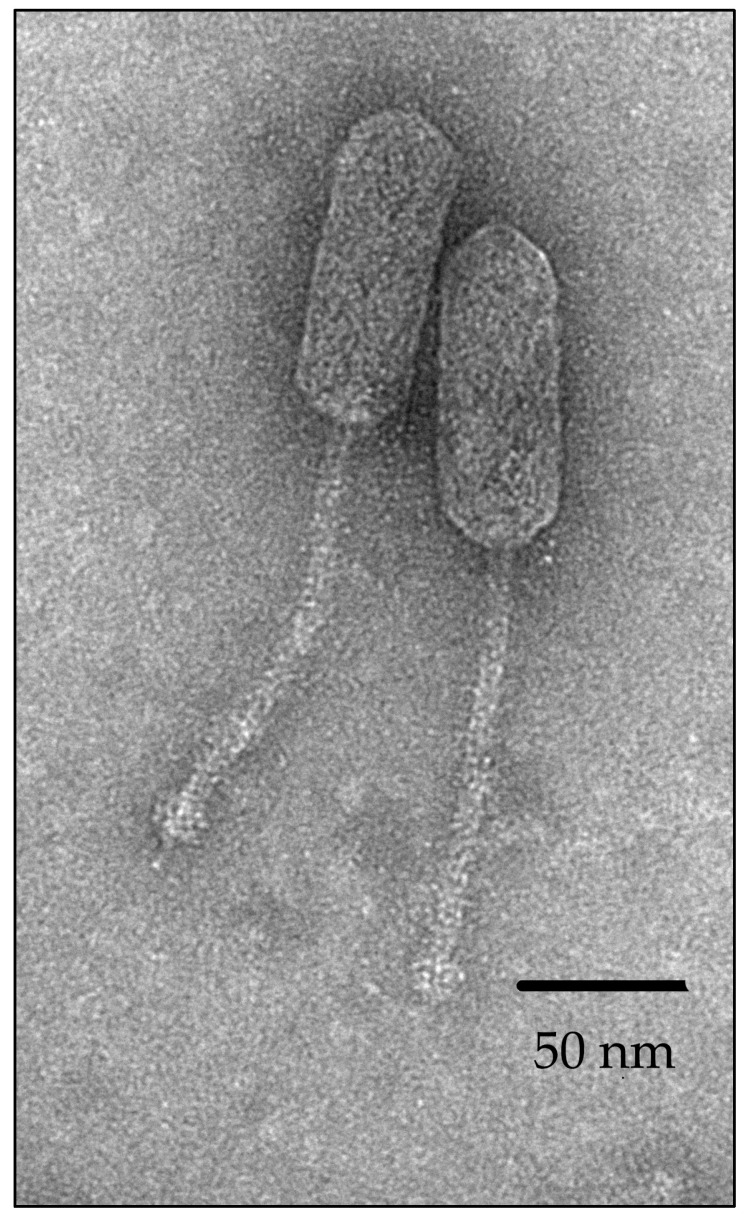
Morphology of bacteriophage vB_EfS_SE virions obtained by transmission electron microscopy.

According to the results of transmission electron microscopy (Figure 1), vB_EfS_SE virions have a siphovirus morphotype. The virions have an elongated capsid 105.2 ± 1.6 nm in length and 39.2 ± 1.6 nm in diameter. The length of the non-contractile tail is 148.4 ± 1.7 nm.

### 3.3. DNA Packing and Chromosome Organization

**Figure 2:**
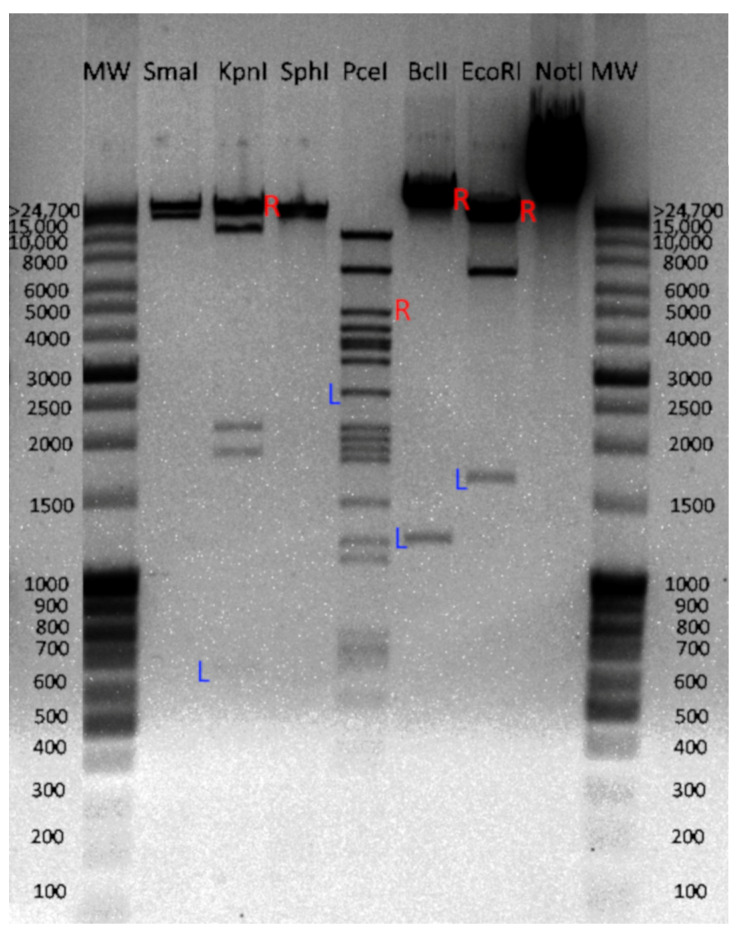
Electropherogram of the vB_EfS_SE chromosome after treatment with restriction endonucleases. Additional fragments containing chromosome ends are marked as L (left) and R (right).

For phages most closely related to vB_EfS_SE, the mechanism of DNA packaging has not been determined experimentally. The chromosome DNA of vB_EfS_SE was digested using restriction endonucleases. In the restriction patterns that were generated with KpnI, PceI, BclI, and EcoRI endonucleases, some of the detected fragments had molecular weights different from the calculated ones. Such deviating fragments contain a restriction endonuclease site on one end of the molecule and the phage chromosome terminus on the other end. Based on the fluorescence intensity of those DNA bands, we established that the fragments were in an equimolar ratio with other fragments (Figure 2), which is typical of phages with direct terminal repeats (DTR) or cohesive sites (cos). DNA fragments obtained by KpnI, BclI, and EcoRI exceeded the calculated size by approximately 300 bp, suggesting that in the phage vB_EfS_SE, DNA packaging most likely occurs through a 300-bp long DTR.

### 3.4. Genome Sequence

**Figure 3:**
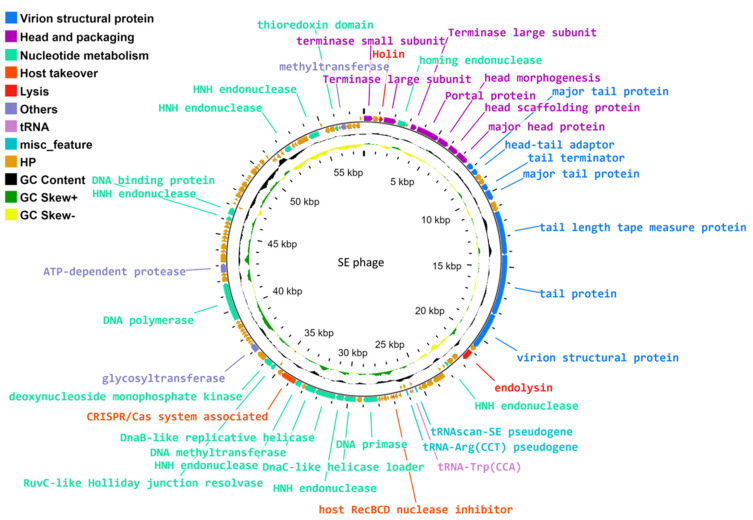
vB_EfS_SE-bacteriophage genome map.

The assembled genome of phage vB_EfS_SE has a length of 57,904 bp. The genome sequence was deposited in NCBI GenBank under accession number PP583004.1. The GC-content is 39.64%. The genome contains 115 protein coding sequences (CDSs) and one tRNA gene (Figure 3).

### 3.5. Genome Organization

The capsid structural gene module contains six genes: portal protein (CDS8), head scaffolding protein (CDS10), major head protein (CDS11), terminase small subunit (CDS1), and terminase large subunit (CDS4, CDS7). The presence of two large subunits is atypical for bacteriophages and, in the case of phage vB_EfS_SE, is possibly due to the presence of a homing endonuclease gene (CDS5) between the large terminase subunit genes (Figure 3, Supplementary Table S1). Alignment of the vB_EfS_SE genome with the genomes of related phages showed that the presence of two large terminase subunit genes is commonly found in related phages (see Section 3.6).

The tail gene module contains major tail protein (CDS12), tail terminator (CDS16), major tail protein (CDS17), tail length tape measure protein (CDS20), and tail protein (CDS21). Predicted endolysin (CDS24) and holin genes (CDS3) are located in different parts of the genome. The following genes of nucleotide metabolism were detected: four HNH endonucleases, methyltransferase, resolvase, deoxynucleoside monophosphate kinase, helicase, helicase loader, primase and DNA polymerase. The genome contains a tRNA-Trp gene and two pseudo-tRNAs. The phage also possesses genes of host suppression: antiviral nuclease inhibitor and CRISPR/Cas-associated gene, endolysin, and holin. No genes of pathogenicity factors or antibiotic resistance were found in the genome.

### 3.6. Phylogenetic Analysis

**Figure 4:**
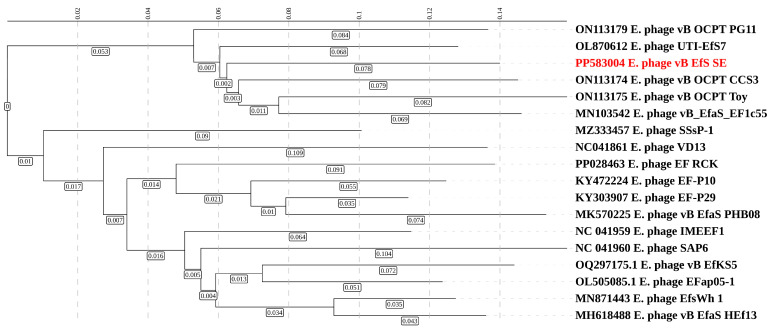
Phylogenetic tree of the vB_EfS_SE phage inferred from a proteome sequences alignment. The branch lengths are shown in the boxes.

A phylogenetic analysis of the translated proteome of vB_EfS_SE, conducted using the VipTree V4.0 software [21], revealed a small group of closely related *Saphexavirus* (Figure 4). The closest was the previously described Enterococcus phage UTI-EfS7. The phage was isolated in Switzerland (Zurich) [22]. Another close relative, Enterococcus phage vB_EfaS_EF1c55, was isolated in Poland.

**Figure 5:**
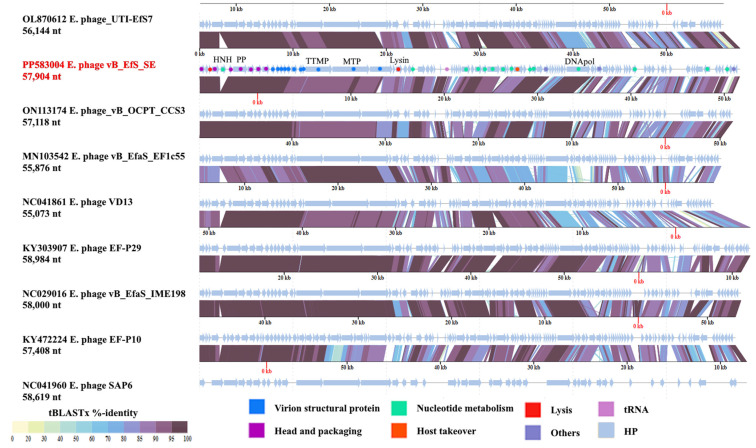
The pairwise tBLASTx whole-genome comparison performed for vB_EfS_SE and related phages.

Using the Viridic V1.1 program [23], we constructed a map of genomic similarity of the phages listed above. Interestingly, the genomes of the related bacteriophages UTI-EfS7 and vB_OCPT_CCS3 contained only two terminase genes. Additionally, in the space between the genes of the small and large subunits of terminase, homing endonuclease was not detected (Figure 5). However, in some more distantly related phages such as Enterococcus phage VD13, the arrangement seen in the vB_BtS_SE phage, with three terminase genes and a homing endonuclease gene, is also present (Figure 5).

### 3.7. Killing Assay, Mg^2+^ and Ca^2+^ Effects

**Figure 6:**
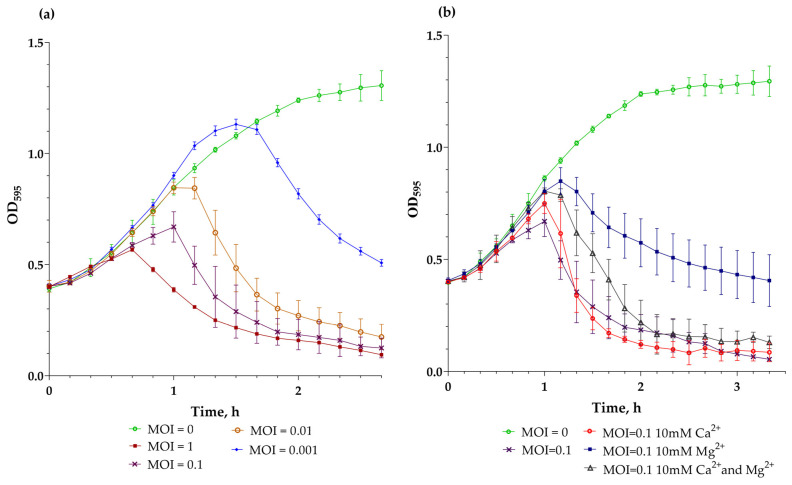
Killing assay at different MOI on *E. faecalis* VKPM B-12629 (**a**) and Ca^2+^/Mg^2+^ effects on vB_EfS_SE-phage lytic properties at MOI = 0.1 (**b**).

The higher the MOI value, the faster the lysis of an vB_EfS_SE-infected culture occurred (Figure 6, t = 0). At MOI = 1, a drop in OD595 was observed after 40 min of incubation. At MOI 0.01–1, the culture was lysed almost completely; however, in the case of MOI = 0.001, the culture lysis was incomplete. Even after 2 h and 40 min of incubation, OD595 remained at around 0.5–0.6. The addition of Ca^2+^ did not significantly affect the lytic curve. The introduction of Mg^2+^ reduced the lytic properties of the bacteriophage. Firstly, a decrease in optical density was observed only at 80 min of incubation as opposed to 60 min when the phage was added without Mg^2+^. Secondly, after 3 h 20 min of incubation, OD595 decreased only to 0.4, as opposed to 0.1 without Mg^2+^. When both Ca^2+^ and Mg^2+^ were added, a decrease in the negative effect of Mg^2+^ was observed. Apparently, Ca^2+^ is required for proper interaction of phage vB_EfS_SE with *E. faecalis* VKPM B-12629.

### 3.8. Thermostability and pH-Stability

**Figure 7:**
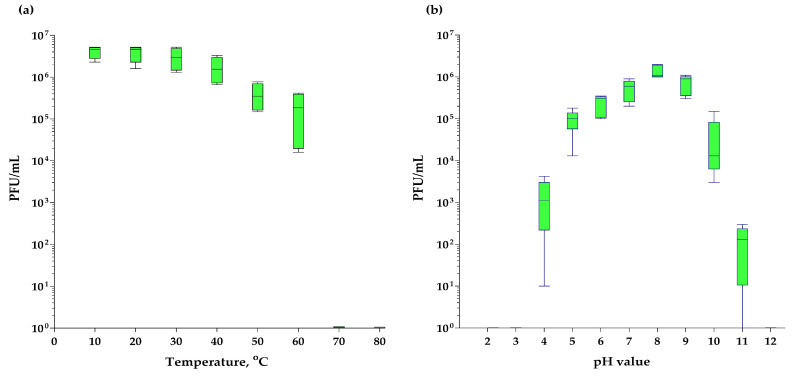
Thermal (**a**) and pH-stability (**b**) of vB_EfS_SE phage virions.

The bacteriophage vB_EfS_SE was completely stable at temperatures from 4 to 30 °C. After incubation for 1 h at 50 °C, the concentration of active virions decreased by 5.5 times, and at 60 °C, by 33 times (Figure 7a). After incubation at 70 °C and higher temperatures, active virions were not detected. When incubated for an hour at different pH values, the phage was stable in the range of 7–9. At pH values of 5 and 10, the number of viable virions decreased by approximately 10 times, and at pH values of 4 and 11, more than 10^3^ times (Figure 7b). After an hour of incubation at even higher and lower pH values, viable virions were not detected.

### 3.9. vB_EfS_SE-Phage Endolysin (PlySE), and the Design and Construction of Its Homologs

To determine the bacteriolytic properties of the vB_EfS_SE endolysin PlySE, its gene was cloned into pBAD18 plasmid and expressed from *E. coli* TOP10 as a recombinant protein. In addition, two catalytic domains (EAD) of close homologues of the PlySE endolysin were synthesized: EAD of the LysIME-EF1 endolysin (AGR49070, 1–143 aa residues), and EAD of the CHAP-domain containing protein from mature sheep metagenome (EGO5065395, 1–142 residues). The resulting proteins contained the catalytic domain (EAD) of the LysIME-EF1 endolysin and a PlySE EAD homolog from a sheep metagenome, as well as the carbohydrate binding domain (CBD) of the vB_EfS_SE endolysin PlySE (168–234 aa) (Supplementary Figure S2). All of the endolysins were purified using metal-chelating chromatography. The purified endolysins contained additional short chains (Supplementary Figure S3) due to the tetrameric organization of CBD.

### 3.10. The Bacteriolytic Spectrum of PlySE Endolysin and Its Homologs

The spectrum of bacteriolytic activity for PlySE, PlyIME-SE, and PlySheep-SE was determined by spot test. All three endolysins showed the same spectrum of bacteriolytic activity, similar to the lytic activity of bacteriophage vB_EfS_SE (Table 1).

### 3.11. pH-Range of Endolysins Activity

**Figure 8:**
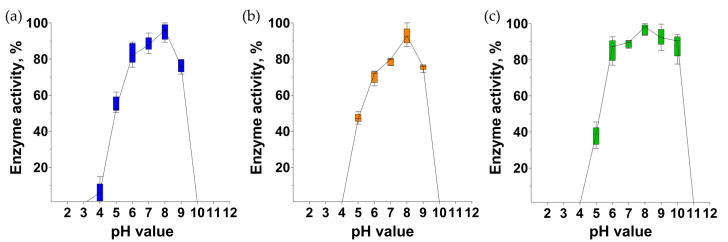
Relative endolysin activity of PlySE (**a**), PlyIME-SE (**b**), and PlySheep-SE (**c**) at different pH-values at 35 °C.

The activity of endolysins was measured at different pH levels. All endolysins retained 75% activity in the pH range from 6 to 9 (Figure 8). At a pH value of 5.0, the activity of all endolysins was about 50% of their maximal activity. The PlySheep-SE endolysin retained 90% of its activity at pH 10, while PlyIME-SE and PlySE were inactive (Figure 8).

### 3.12. Endolysins Thermostability

**Figure 9:**
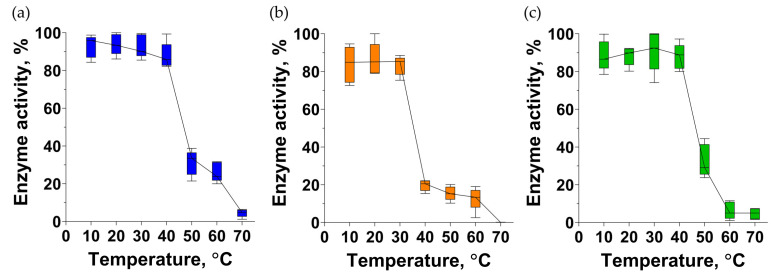
Thermostability of endolysins PlySE (**a**), PlyIME-SE (**b**) and PlySheep-SE (**c**).

PlyIME-SE retained about 20% of activity after 1 h of incubation at 40 °C (Figure 9). PlySE and PlySheep were completely intact at 40 °C, but retained only 30% of activity after being incubated at 50 °C. To completely inactivate each of the endolysins, 1 h of incubation at 70 °C was required (Figure 9).

## 4. Discussion

Bacteriophages with a high similarity to vB_EfS_SE have been previously isolated from various sources in China, South Korea, Poland, Russia, the USA, Japan, and Malaysia [24,25,26,27,28,29]. They belong to the genus *Saphexavirus* and have a latent period of 18 (SSsP-1) to 30 min (EF-P29). The progeny size varies from 66 (SSsP-1, WH1) to 352 (vB_EfaS_HEf13) [26,27,28,29,30]. The described representatives of *Saphexavirus* are stable at neutral pH and can decay significantly in a weakly acidic and slightly alkaline pH range [24,26,27,30,31]. Phage vB_EfaS_HEf13 virions were shown to remain stable over the widest pH range from 3 to 11, without undergoing significant concentration decrease [26]. All reports have shown that *Saphexavirus* virions remain intact at 40 °C [24,27,30], and at least vB_EfaS_HEf13 [26] and vB_EfaS_efap05-1 [31] did not detectably decay even at 50 °C. Complete elimination of *Saphexavirus* phages usually requires 1 h incubation at 70 °C [24,26,30,31], with one exception: the vB_EfKS5 phage can only be eliminated at 80 °C [27]. The fraction of sensitive strains varies from less than 15% [25] to 50–70% [24,26,30,31].

Bacteriophages of the genus *Saphexavirus* have shown success in decontaminating food in model experiments. The vB_EfKS5 phage can inactivate *E. faecalis* in milk, with a 10^5^-fold reduction when acting alone, and a 10^6^-fold reduction in the presence of nisin [27]. The vB_EfaS_WH1 phage reduced a concentration of *E. faecalis* on the surface of chicken breast meat a few hundred-fold [30]. The bacteriophages IME-EF1, EF-P29, SSsP-1, and φ45 can save mice from sensitive strains of *E. faecalis* when applied alone [28,29,30] or as a part of a phage cocktail [32].

In a mouse model, it was shown that bacteriophage φ45 (ON086985, 93% identity, 81% coverage with vB_EfS_SE) has reduced immunogenicity compared to phages of the genus *Kochikohdavirus* [32]. Homologues of the genes encoding proteins that caused an immune response upon introduction of phage φ45 are present in the genome of vB_EfS_SE: phage tail fiber protein (putative tail protein, XBC18204, 68.67% identity, 100% coverage with YP_009604020.1); phage minor structural protein (virion structural protein, XBC18205, 92.43% identity, 55% coverage with YP_009604019.1); and portal protein (XBC18191 99.60% identity, 96% coverage with QEM41711.1) [32]. An in silico structural model was obtained of a minor structural protein homolog of vB_EfaS_TV16 (vB_EfS_SE: XBC18205) [33]. The model was based on 29% amino acid sequence identity with streptococcal phage-encoded tail-fiber with hyaluronan lyase activity (PDB 2C3F). However, there is no direct experimental evidence of its activity, except that the vB_EfaS_PHB08, vB_EfKS5, and vB_EfaS_WH1 phages showed the ability to eradicate biofilms [24,27,30].

Among the representatives of the *Saphexavirus* genus, no temperate phages or genes responsible for lysogenic state maintenance were found. Resistance to these bacteriophages can develop due to mutations in receptors responsible for the adsorption of the phage virions on the bacterial cell surface. Thus, in mutant strains of *E. faecalis* that have developed resistance to vB_EfaS_efap05-1, mutations were found in two genes: *com*EA, responsible for bacterial competence, and UDP-glucose 4-epimerase *gal*E, responsible for surface polysaccharide biosynthesis. This suggests that the vB_EfaS_efap05-1 phage uses either surface polysaccharide or membrane protein ComEA as the receptor for adsorption on the bacterial wall surface [31].

Among close homologs (>59% identity, 100% coverage) of the PlySE endolysin, a number of endolysins were studied: LysIME-EF1 (YP_009603970) [25], LysEF-P10 (AQT27695) [34], V13 endolysin (YP_009036394) [35], and Lys08 (QBX32948) [24]. Endolysins usually have a wider range of bacteriolytic activity in comparison with phages. The LysIME-EF1 endolysin was active against 10 of the 10 *E. faecalis* strains tested, while its source phage, IME-EF1, was active against only 3 of them. Both the IME-EF1 phage and its endolysin were active against 1 out of 10 *E. faecium* strains [25]. LysEF-P10 was active against 32 of 36 vancomycin-resistant *E. faecalis* strains, but did not target any of the six *E. faecium* strains tested [34]. The endolysin of the VD13 phage lysed 10 of 12 *E. faecalis* strains, while the phage itself was able to lyse only 5 [35]. In our work, two synthetic endolysins were constructed. The first one, PlyIME-SE, consists of the EAD from the LysIME-EF1 endolysin [25] and CBD from the SE-phage endolysin. The second endolysin molecule, PlySheep-SE, was constructed in the same way and consists of an EAD homolog sequence from a sheep microbiome and a CBD from the SE-phage endolysin. The lytic spectrums of the PlySE, PlyIME-SE, and PlySheep-SE endolysins were identical. The endolysins lysed only one out of three *E. faecalis* strains tested, but were active against *E. faecium* (one of eleven strains), *E. avium* (one of one strains), and *E. hirae* (one of one strains) (Table 1). Our collection did not contain vancomycin-resistant strains or clinical isolates, which can explain the narrow specificity of the constructed endolysins and the vB_EfS_SE.

PlySE, PlyIME-SE, and PlySheep-SE showed maximum activity in a neutral range, and lost more than 50% of activity at pH 5. In contrast, the activity of the VD13 phage endolysin was maximal at pH 4 and 5, while at pH levels ranging from 6 to 10, it was at least three times lower than at pH 5.

The endolysin of VD13 has been studied for thermostability: at pH 7, the endolysin lost its bacteriolytic activity at 42 °C, whereas at pH 5, inactivation occurred only at 65 °C. [35]. In our work, the thermostability experiments were conducted at pH 7 (Figure 9). The activity of PlySE and PlySheep-SE decreased between 40 and 50 °C, and PlyIME-SE became partially inactivated between 30 and 40 °C. Interestingly, all three endolysins studied in this work retained up to 25% of activity even after 1 h incubation at 60 °C, and became completely inactive after 1 h heating at 70 °C.

For one of the closest PlySE homologs, LysIME-EF1 of the Enterococcus phage IME-EF1 (NC_041959.1), a 3D structure was solved using X-ray analysis [20]. LysIME-EF1 (PDB 6IST) has a heterotetrameric structure. It consists of one 237-aa subunit with two domains: an enzyme activity domain (EAD, 1–143 aa), and a carbohydrate binding domain (CBD, 168–237 CBD), as well as three other short subunits with only a CBD domain. Short endolysin subunits are produced when ribosomes recognize an alternative Shine–Dalgarno sequence located near methionine codon M168 in the mRNA [20].

Interestingly, when LysEF-P10 was expressed with the EAD or CBD domain deleted, the truncated variants did not exhibit bacteriolytic activity. However, CBD LysEF-P10 or CBD fused to green fluorescent protein retained its cell wall binding activity [34]. Similar results were obtained for the LysIME-EF1 F184R/Y218A mutant. This mutant is unable to form a tetramer, resulting in a lack of bacteriolytic activity [20].

A BLASTp search for PlySE CBD (168–237) homologs revealed a number of homologs among bacteriophages with morphotype podovirus. One of the homologs (74.29% identity, 100% coverage) is endolysin (YP_009191336) of the bacteriophage vB_EfaP_IME195. The vB_EfaP_IME195 phage has an icosahedral capsid of ~41 nm in diameter, and its genome length is 18,607 bp [36]. The predicted activity of the N-terminal EAD of its endolysin is N-acetylmuramoyl-L-alanine amidase, and its sequence has no homology with the PlySE EAD.

Considering that (i) substitution of EAD in PlySE molecule does not lead to a change in specificity (Table 1); (ii) tetrameric type CBDs are common among enterococcal bacteriophages with siphovirus (*Saphexavirus*) and podovirus (vB_EfaP_IME195) morphotypes; and (iii) an individual EAD without CBD or mutant CBD lacking multimerization causes a loss of lytic activity [20,34], it can be assumed that CBD determines the specificity of endolysins homologous to PlySE, while EAD determines the particularities of the catalytic activity.

## 5. Conclusions

The bacteriophages described in this work usually infect no more than half of the tested enterococci strains, and are not active against streptococci, staphylococci, and other bacteria. For this reason, it is sensible to use them in cocktails containing 3–5 phages. Taking into account the low immunogenicity of phages, we can highly estimate the potential of *Saphexavirus* phages as part of therapeutic phage cocktails.

Endolysins based on the PlySE molecule may become the basis for the development of new antibacterial molecules. They have been shown to protect mice when administered 30 min after infection [25]. Five micrograms per mouse admission saved the animals from vancomycin-resistant *E. faecalis*. The antibodies concentration reached a maximum 3 weeks after injecting LysEF-P10, but after eight weeks, the anti-LysEF-P10 antibodies become undetectable. The LysEF-P10 remained active in the presence of antisera from the immunized mice [34]. However, to fully realize the potential of PlySE-like endolysins, it is necessary to investigate how they interact with its receptor on the bacterial cell.

## Figures and Tables

**Table 1:** *Enterococcus* strain sensitivity to the vB_EfS_SE phage and endolysins.

#	Species	Strain	vB_EfS_SE	PlySE	PlyIME-SE	PlyShip-SE
1	*E. avium*	VKM B-1673	+	+	+	+
2	*E. durans*	VKM B-603	-	-	-	-
3		VKPM B-8257	-	-	-	-
4		VKPM B-11854	-	-	-	-
5	*E. faecalis*	VKPM B-12629	+	+	+	+
6		VKPM B-4426	-	-	-	-
7		VKPM B-4053	-	-	-	-
8	*E. faecium*	FS86	-	-	-	-
9		VKPM B-2579	-	-	-	-
10		VKPM B-2990	-	-	-	-
11		VKPM B-3490	-	-	-	-
12		VKPM B-3491	-	-	-	-
13		VKPM B-4054	+	+	+	+
14		VKPM B-4489	-	-	-	-
15		VKPM B-4991	-	-	-	-
16		VKPM B-5000	-	-	-	-
17		VKPM B-8551	-	-	-	-
18		VKPM B-12648	-	-	-	-
19	*E. hirae*	VKPM B-12152	+	+	+	+
20	*E.* sp.	VKM B-1578	-	-	-	-
21		VKM B-1944	-	-	-	-
22		VKPM B-3371	-	-	-	-
23		VKPM B-8711	-	-	-	-
24		VKPM B-8712	-	-	-	-
25		VKPM B-8713	-	-	-	-
26	*E. thailandicus*	VKPM B-10684	-	-	-	-
	*E. coli*	XL1 Blue	-	-	-	-
	*B. cereus*	B370	-	-	-	-

## Data Availability

The annotated complete genome of the vB_EfS_SE bacteriophage was deposited into GenBank under accession number PP583004.

## Supplementary Materials

The following supporting information can be downloaded at: https://www.mdpi.com/article/10.3390/pharmaceutics16101312/s1. Figure S1. The morphology of negative colonies of vB_EfS_SE-phage *Enterococcus hirae* VKPM B-12152 lawn. Figure S2. The scheme of chimeric endolysins. Figure S3. SDS-PAGE assay of purified endolysins fractions PlySE (track 1, 28.4 kDa); PlyIME-SE (track 2 ,28.4 kDa); and PlySheep-SE (track 3, 28.7 kDa). Table S1. Annotation of *Enterococcus phage* vB_EfS_SE genome. Table S2. Oligonucleotide primers used for cloning the PlySE, PlyIME-SE and PlySheep-SE endolysin genes.
