# Supplementary material for: Characteristics of the Enterococcus Phage vB_EfS_SE, and the Properties of Its Chimeric Endolysins Harboring a PlySE-Carbohydrate-Binding Domain and a Synthetic Enzymatic Domain
Source: Pharmaceutics. 2024 Oct 9;16(10):1312. doi: 10.3390/pharmaceutics16101312 (PMC11510935; doi:10.3390/pharmaceutics16101312)
Supplement: Supplementary file 1 [file pharmaceutics-16-01312-s001.zip › pharmaceutics-3201334-supplementary.pdf]

Supplementary Information:

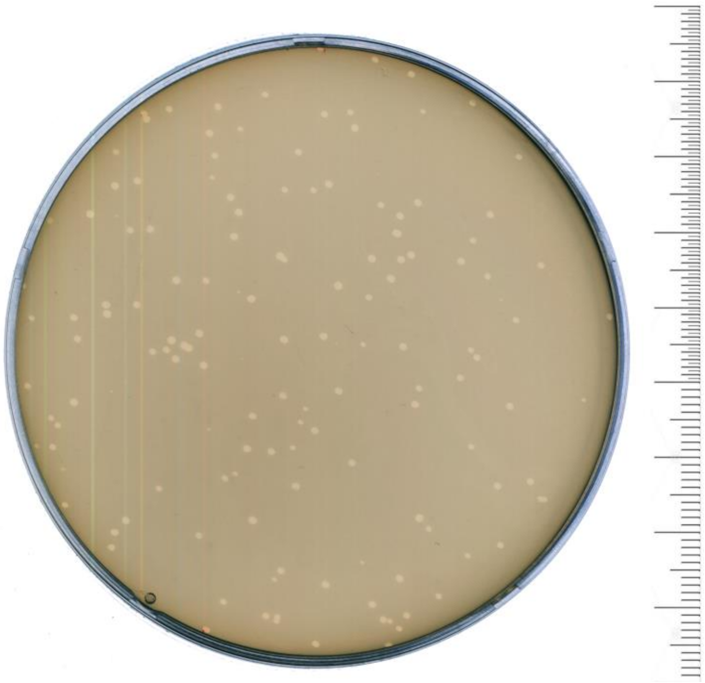

Supplementary figure S1. The morphology of negative colonies of vB_EfS_SE-phage *Enterococcus hirae* VKPM B-12152 lawn.

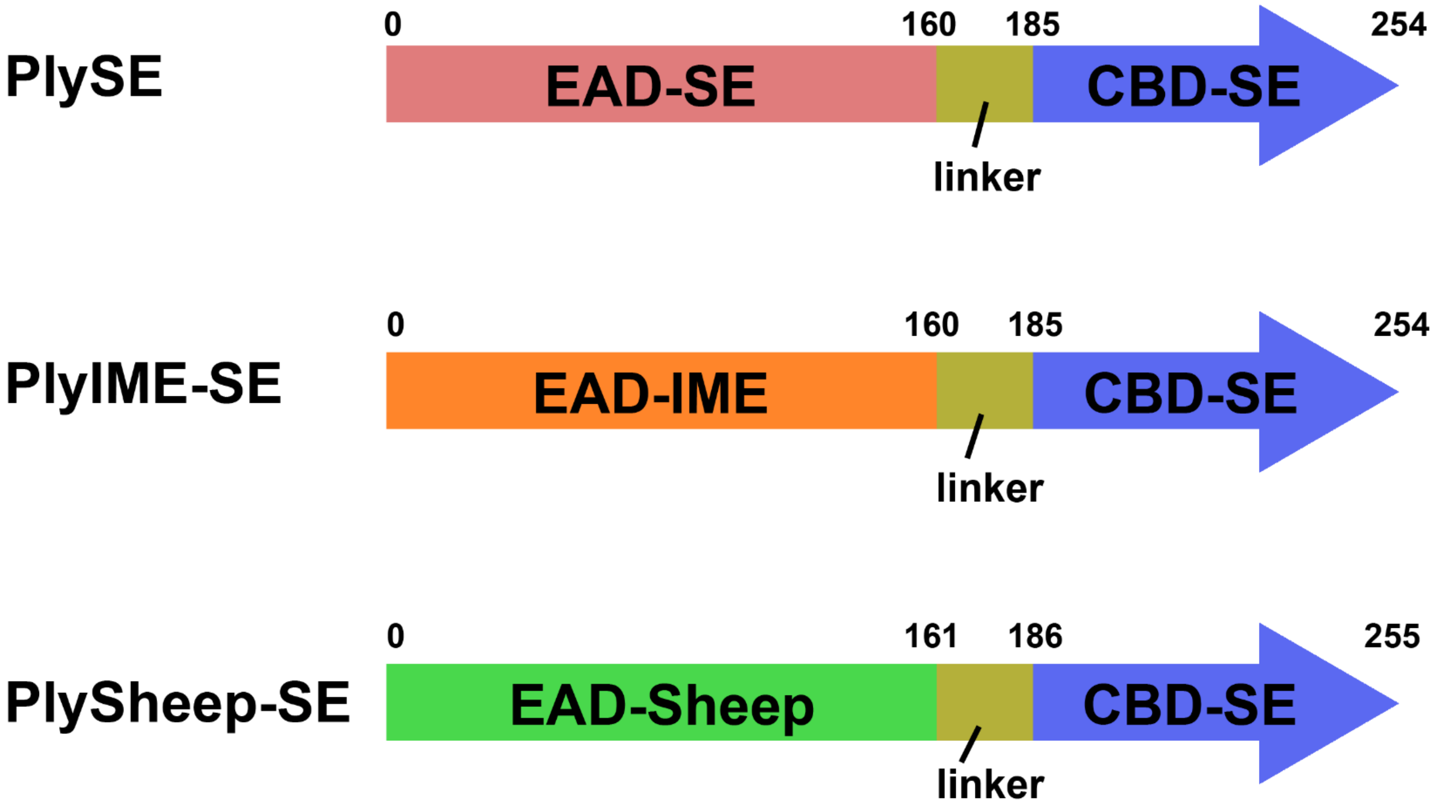

Supplementary Figure S2. The scheme of chimeric endolysins.

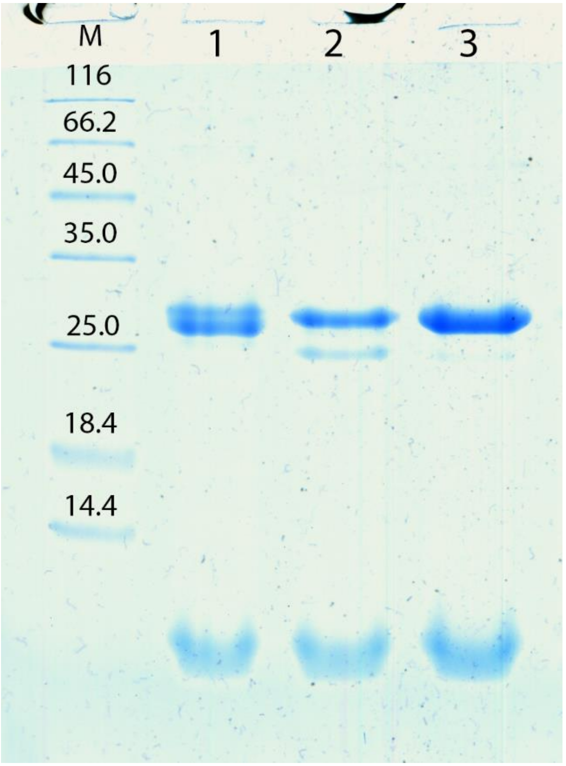

Supplementary Figure S3. SDS-PAGE assay of purified endolysins fractions PlySE (track 1, 28.4 kDa); PlyIME-SE (track 2 ,28.4 kDa); and PlySheep-SE (track 3, 28.7 kDa).

Supplementary Table S1. Annotation of Enterococcus phage vB_EfS_SE genome

ORF#	Start codon	Stop codon	Strand	Blast results		Conserved domains, Blast		Hhpred results	Annotation
				Name	E-val	Name, (region)	E-val	(Prob./E-val)	
1	1	597	+	terminase small subunit [Enterococcus phage VD13]	2e-137	Terminase_2 - Terminase small subunit; (34-150)	1.84e-11	Terminase small subunit; DNA-binding protein, DNA pakaging, Bacillus phage SF6 / 2.2e-15	Terminase small subunit
2	612	989	+	hypothetical protein X878_0012 [Enterococcus phage VD13]	9e-52	-	-	DUF 2951; Protein of unknown function / 0.51	hp
3	1008	1256	+	holin [Enterococcus phage VD13]	2e-41	-	-	Phage_holin_5_1; Bacteriophage A118-like holin, Hol118 / 8.7e-19	Holin
4	1319	2122	+	terminase large subunit [Enterococcus phage BC611]	0.0	Terminase_3, Phage terminase large subunit; (29-226)	1.65e-67	Large subunit terminase; large terminase, VIRAL PROTEIN; 2.2A Deep-sea thermophilic phage D6E / 4.7e-28	Terminase large subunit
5	2253	2855	+	homing endonuclease [Enterococcus phage VD13]	2e-126	grpIintron_endo group I intron endonuclease, (3-190)	4.48e-25	SegE_GIY-YIG; Putative endonuclease segE, GIY-YIG domain / 3.2e-12	Homing endonuclease
6	2836	2943	+	-	-	-	-	DUF2996; Protein of unknown function / 43	hp
7	3140	3529	+	Terminase large subunit [Enterococcus phage VD13]	6e-88	Terminase_3C, Terminase RNAseH like domain, (8-110)	6.81e-18	Terminase_3C; Terminase RNAseH like domain / 1.5e-14	Terminase_3C or Terminase large subunit

8	3588	5123	+	portal protein [Streptococcus phage SP-QS1]	0.0	Phage_prot_Gp6, Phage portal protein, SPP1 Gp6-like, (40-456)	2.07e-81	Portal protein; Bacteriophage, SPP1, Portal Protein, Head completion proteins, Connector Complex, DNA Channel, VIRAL PRO / 1.1e-37	Portal protein
9	5135	5890	+	head morphogenesis [Enterococcus phage BC611]	3e-163	-	-	COG2369; Uncharacterized conserved protein, contains phage Mu gpF-like domain [Function unknown] / 1.5e-18	hp
10	6001	6669	+	head scaffolding protein [Enterococcus phage vB_EfaS_IME198]	9e-152	DUF4355, Domain of unknown function, (60-171)	3.34e-06	Scaffold protein; major capsid protein, HK97-like fold, scaffolding protein, procapsid, VIRUS; 3.72A [Staphylococcus phage] / 4.4e-16	head scaffolding protein
11	6718	7524	+	major head protein [Enterococcus phage vB_EfaS_IME198]	0.0	major capsid protein, N4-gp56 family, (6-132)	9.34e-15	major capsid protein; acne, bacteriophage, HK97-like, VIRUS; 3.7A {Propionibacterium phage PA6} / 8.4e-25	major head protein
12	7679	8116	+	major tail protein [Streptococcus phage SP-QS1]	3e-92	Uncharacterized conserved protein YjdB, contains Ig-like domain, (61-144)	6.75e-08	b.1.2.1 (A:8-124) Rim binding protein 2 {Human (Homo sapiens) [TaxId: 9606]} | CLASS: All beta proteins, FOLD: Immunoglo / 20.93	hp
13	8177	8581	+	hypothetical protein EnPhBC-611_gp49 [Enterococcus phage BC611]	3e-95	-	-	Head completion protein gp15; Bacteriophage, SPP1, Portal Protein, Head completion proteins, Connector Complex, DNA Chan / 9e-15	hp

14	8597	9874	+	hypothetical protein EnPhBC-611_gp48 [Enterococcus phage BC611]	4e-80	-	-	b.1.2.1 (R:2-100) Interleukin-10 receptor 1, IL-10R1 {Human (Homo sapiens) [TaxId: 9606]} | CLASS: All beta proteins, FO / 270	hp
15	8959	9339	+	hypothetical protein BN19_012 [Streptococcus phage SP-QS1]	8e-75	-	-	COG5005; Mu-like prophage protein gpG [Mobilome: prophages, transposons]. / 4.6e-15	hp
16	9352	9786	+	tail terminator [Streptococcus phage SP-QS1]	4e-102	DUF3168 Protein of unknown function, (30-106)	1.87e-05	Tail terminator protein Rcc01690; "neck", "portal", "capsid", "tail tube", VIRUS; 3.58A {Rhodobacter capsulatus} / 1.8e-17	Tail terminator protein
17	9807	10499	+	major tail protein [Enterococcus phage IMEEF1]	5e-166	Phage_tail_2, Phage tail tube protein, (9-135)// Big_2, Bacterial Ig-like domain (group 2), (154-215)	6.07e-26 //4.11e-08	Tail tube protein; Tail tube, siphoviridae, helical, VIRAL PROTEIN; 5.4A {Escherichia phage lambda} / 7e-15	Tail tube protein
18	10639	11079	+	hypothetical protein AVT94_gp38 [Enterococcus phage vB_EfaS_IME198]	3e-100	-	-	DUF5055; Domain of unknown function / 5.7e-14	hp
19	11018	11314	+	hypothetical protein BN19_016 [Streptococcus phage SP-QS1]	2e-45	-	-	Phage_TAC_6; Phage tail assembly chaperone protein, TAC / 49.02	hp
20	11327	14212	+	tail length tape measure protein [Enterococcus phage BC611]	0.0	tape_meas_TP901, phage tail tape measure protein,	4.56e-58	Tape Measure Protein, gp57; phage tail, tail tip, tape measure protein, VIRAL PROTEIN;	tail length tape measure protein

						TP901 family, core region; (97-432)		3.7A {Staphylococcus virus 80alph} / 3.3e-15	
21	14226	18218	+	tail protein [Enterococcus phage IMEEF1]	0.0	-	-	Tail component; bacteriophage infection, Lactobacillus casei / 3.9e-23	Putative tail protein
22	18231	20681	+	virion structural protein [Enterococcus phage IMEEF1]	0.0	put_anti_recept, phage minor structural protein, N-terminal region; (31-359)	6.24e-13	PblB; Phage-related protein [Mobilome: prophages, transposons] / 2.1e-23	virion structural protein
23	20629	20982	+	hypothetical protein X878_0032 [Enterococcus phage VD13]	1e-61	-	-	DUF2977; Protein of unknown function / 0.0051	hp
24	21031	21744	+	endolysin [Enterococcus phage VD13]	2e-158	CHAP domain, (20-110)	2.61e-06	Lysin; Lysin, HYDROLASE; 1.75A {Enterococcus phage IMEEF1}/1.2e-28	endolysin
25	22194	21793	-	hypothetical protein AVT94_gp45 [Enterococcus phage vB_EfaS_IME198]	5e-20	-	-	a.24.30.1 (A:504-592) CRISPR-Cas type III-B interference complex protein Cmr2, D2 {Pyrococcus furiosus [TaxId: 2261]} |/ 30.33	hp
26	22655	22227	-	hypothetical protein AVT94_gp46 [Enterococcus phage vB_EfaS_IME198]	3e-93	-	-	60 kDa polyprotein; VIRAL PROTEIN; 1.9286A {African swine fever virus / 29.33	hp
27	22881	22657	-	NUMOD4 domain-containing protein [Clostridium perfringens]	1e-06	IENR1, Intron encoded nuclease repeat motif, (32-66)	1.87e-03	HNH homing endonuclease; HNH catalytic motif, Helix-turn-helix DNA binding domain, protein-DNA complex, DNA binding protein / 0.012	HNH homing endonuclease

28	23101	22973	-	-	-	-	-	COG3803; Uncharacterized conserved protein, DUF924 family [Function unknown]. / 22.15	hp
29	23450	23325	-	-	-	-	-	DUF1764; Eukaryotic protein of unknown function (DUF1764) / 18	hp
30	23380	24204	+	hypothetical protein AVT94_gp47 [Enterococcus phage vB_EfaS_IME198]	0.0	-	-	gp32 single stranded DNA binding protein; Zn2+ binding subdomain, 5-stranded beta-sheet, OB fold / 0.0000011	hp
31	24694	25080	+	hypothetical protein AVT94_gp49 [Enterococcus phage vB_EfaS_IME198]	7e-90	-	-	Minimized B-domain of Protein A Z34C; IgG1 fc, protein A, fc complex, b-domain, IMMUNE SYSTEM; HET: BMA, MAN, GAL, FUL,/ 33	hp
32	25080	25280	+	hypothetical protein AVT94_gp50 [Enterococcus phage vB_EfaS_IME198]	3e-40	-	-	b.34.20.1 (A:7-71) Uncharacterized protein YorP {Bacillus subtilis [TaxId: 1423]} | CLASS: All beta proteins, FOLD: SH3- / 1.4	hp
33	25678	25562	-	-	-	-	-	Ly49 ; Ly49-like protein, N-terminal region / 7.7	hp
34	25779	25904	+	hypothetical protein BN19_031 [Streptococcus phage SP-QS1]	3e-17	-	-	Transmembrane protein, putative; Electron transport chain, supercomplex, membrane protein, Tetrahymena thermophila, ELEC / 17	hp
35	26183	26016	-	-	-	-	-	Eco57I; Eco57I restriction-modification methylase / 73	hp

36	26388	26287	-	-	-	-	-	DUF3565; Protein of unknown function (DUF3565) /17	hp
37	26486	26659	+	-	-	-	-	Interleukin-6; CYTOKINE, INTERLEUKIN, SIGNALING, GP-130, TRANSCRIPTION; NMR {Mus musculus} SCOP: a.26.1.0/ 34	hp
38	26729	26929	+	-	-	-	-	Beta-lactamase; colibactin peptidase, S12 peptidase, HYDROLASE, HYDROLASE-INHIBITOR complex; HET: 2PE, Z9A, Z9G, 97N, AV / 10	hp
39	26942	27088	+	host RecBCD nuclease inhibitor [Enterococcus phage IMEEF1]	2e-23	PHA00442, host recBCD nuclease inhibitor (13-47)	9.85e-04	DUF6388; Family of unknown function / 11	hp
40	27069	27233	+	hypothetical protein FDH83_gp78 [Enterococcus phage IMEEF1]	7e-19	-	-	PrgA; Protease domain, CAP domain, CELL ADHESION; HET: EPE; 1.5A {Enterococcus faecalis} / 0.48	hp
41	27328	27465	+	hypothetical protein AVT94_gp53 [Enterococcus phage vB_EfaS_IME198]	8e-22	-	-	hypothetical protein yoaG; Alpha/Beta protein, homodimer, OCSP, NESG, PROTEIN STRUCTURE INITIATIVE, structural genomics, / 0.14	hp
42	27465	27719	+	hypothetical protein FDH83_gp77 [Enterococcus phage IMEEF1]	9e-55	-	-	APC1_3rd; APC1 beta sandwich domain / 95	hp
43	27716	27874	+	hypothetical protein X878_0045 [Enterococcus phage VD13]	4e-24	-	-	DUF6489; Family of unknown function / 4.1	hp
44	27874	28062	+	hypothetical protein AVT94_gp55 [Enterococcus phage vB_EfaS_IME198]	8e-37	-	-	DUF4926; Domain of unknown function / 42	hp

45	28074	29018	+	DNA primase [Enterococcus phage BC611]	0.0	TOPRIM_primases, nucleotidyl transferase/hydrolase domain (204-287)	1.99e-11	DnaG; DNA primase (bacterial type) / 6e-32	DNA primase
46	29093	29446	+	transcriptional regulator [Enterococcus phage IMEEF1]	2e-77	-	-	Putative uncharacterized protein; DNA BINDING PROTEIN; NMR {Hyperthermus butylicus} / 3.6e-8	hp
47	29495	30271	+	DnaC-like helicase loader [Enterococcus phage BC611]	0.0	DnaC, DNA replication protein DnaC, (97-256)	1.09e-09	Primosomal protein DnaI; primase, helicase loader, DnaB, DnaG, DnaI, DNA replication, REPLICATION; HET: MSE; 6.1A / 2.8e-20	DnaC-like helicase loader
48	30858	32201	+	Replicative DNA helicase [Enterococcus phage IMEEF1]	0.0	DnaB, Replicative DNA helicase	3.52e-10	DnaB-like replicative helicase; phage, complex, helicase, REPLICATION-DNA-RNA complex; HET: AGS;{Escherichia phage T4} / 3.6e-39	Replicative DNA helicase
49	32214	32966	+	DNA methyltransferase [Streptococcus phage SP-QS1]	0.0	Cyt_C5_DNA_methylase, Cytosine-C5 specific DNA methylases, (59-149)	1.65e-05	C-5 cytosine-specific DNA methylase [Transcription] / 2.6e-18	DNA methyltransferase
50	32932	33156	+	hypothetical protein EnPhBC-611_gp73 [Enterococcus phage BC611]	5e-44	-	-	VPS38; Vacuolar protein sorting 38 / 26	hp

51	33229	33669	+	HNH endonuclease [Streptococcus phage SP-QS1]	1e-102	HNH endonuclease (60-103)	8.46e-07	HNH homing endonuclease; HNH catalytic motif, Helix-turn-helix DNA binding domain / 3.2e-20	HNH homing endonuclease
52	33662	34690	+	CRISPR/Cas system associated [Enterococcus phage vB_EfaS_IME198]	0.0	-	-	Uncharacterized protein; Cas4, CRISPR, MCSG, Exonuclease, PSI-Biology, STRUCTURAL GENOMICS, UNKNOWN FUNCTION / 8.1e-13	Putative CRISPR/Cas system associated
53	34690	35019	+	hypothetical protein X878_0053 [Enterococcus phage VD13]	8e-70	-	-	TRANSCRIPTION INITIATION FACTOR IIE SUBUNIT ALPHA, TFA1; TRANSCRIPTION, PRE-INITIATION COMPLEX, RNA POLYMERASE / 7	hp
54	35142	35005	-	-	-	-	-	WWamide peptide / 24	hp
55	35285	35590	+	crossover junction endodeoxyribonuclease RuvC [Enterococcus phage IMEEF1]	2e-64	Crossover junction endodeoxyribonuclease RuvC, (11-66)	2.94e-11	RuvC; Holliday junction resolvasome RuvABC endonuclease subunit / 2.6e-9	RuvC-like Holliday junction resolvase
56	35587	36156	+	AAA domain protein [Enterococcus phage VD13]	1e-98	AAA_18, AAA domain, (7-141)	1.46e-03	Phosphomevalonate kinase; parallel beta-sheet with the strand order 23145, Walker A motif / 1.3e-15	monophosphate kinase
57	36149	36781	+	hypothetical protein EnPhBC-611_gp20 [Enterococcus phage BC611]	8e-104	-	-	RNA polymerase sigma-H factor; Sigma factor, / 1.5e-16	hp
58	36879	37442	+	glycosyltransferase	8e-108	-	-	Transcriptional regulatory protein RcsB; Response	hp

								regulator Transcriptional factor / 0.23	
59	37442	37552	+	-	-	-	-	Vpu protein / 3.3	hp
60	37568	37747	+	hypothetical protein FDH83_gp59 [Enterococcus phage IMEEF1]	9e-16	-	-	fvmX3; FtsH ternary system domain X3 / 21	hp
61	37749	38018	+	hypothetical protein BN19_054 [Streptococcus phage SP-QS1]	0.001	-	-	Putative salt-induced outer membrane protein YdiY / 12	hp
62	38032	38253	+	hypothetical protein X878_0058 [Enterococcus phage VD13]	4e-42	-	-	TFA2 Winged helix domain 2 / 1.3	hp
63	38253	38462	+	hypothetical protein AVT94_gp73 [Enterococcus phage vB_EfaS_IME198]	6e-37	-	-	Protein Cue2 hypothetical protein YKL090W / 48	hp
64	38465	38677	+	hypothetical protein AVT94_gp74 [Enterococcus phage vB_EfaS_IME198]	4e-39	-	-	d.58.18.6 (A:105-187) Acetolactate synthase small subunit, IlvH / 0.4	hp
65	38678	39055	+	hypothetical protein X878_0060 [Enterococcus phage VD13]	2e-38	-	-	Protein ymcA; ymcA, regulate community development, Structural Genomics, PSI-2, / 0.57	hp
66	39059	39244	+	hypothetical protein EnPhBC-611_gp67 [Enterococcus phage BC611]	5e-35	-	-	DUF5495; Family of unknown function / 3.3	hp
67	39323	41854	+	DNA polymerase [Enterococcus phage vB_EfaS_IME198]	0.0	PolA, DNA polymerase I - 3'-5' exonuclease and polymerase domains, (107-774)	1.24e-86	PolA; DNA polymerase I - 3'-5' exonuclease and polymerase domains / 4.4e-80	DNA polymerase
68	41930	42334	+	hypothetical protein BN19_061 [Streptococcus phage SP-QS1]	1e-19	-	-	c.1.18.0 (A:) automated matches {Thermococcus kodakarensis [TaxId: 69014]} | CLASS:	hp

								Alpha and beta proteins (a/b) / 120	
69	42324	42554	+	hypothetical protein FDH85_gp21 [Enterococcus phage SAP6]	2e-11	-	-	RNA polymerase II mediator complex subunit 20; beta barrel, channel / 47	hp
70	42547	43143	+	ATP-dependent protease [Enterococcus phage vB_EfaS_IME198]	2e-14	-	-	AtpI2; FoF1-type ATP synthase assembly protein I / 7	hp
71	43199	43840	+	Putative cytidine deaminase [Streptococcus phage SP-QS1]	4e-118	-	-	YorP protein / 0.059	hp
72	43852	44058	+	hypothetical protein BN19_066 [Streptococcus phage SP-QS1]	2e-36	-	-	c.37.1.0 (A:316-647) automated matches {Escherichia coli [TaxId: 562]} | CLASS: Alpha and beta proteins / 9.8	hp
73	44072	44485	+	hypothetical protein BN19_067 [Streptococcus phage SP-QS1]	5e-52	-	-	mRNA interferase toxin HicA; toxin-antitoxin, TA, protein complex, DNA-binding / 26	hp
74	44563	44751	+	hypothetical protein BN19_068 [Streptococcus phage SP-QS1]	2e-31	-	-	YtcA family / 11	hp
75	44751	45008	+	hypothetical protein BN19_070 [Streptococcus phage SP-QS1]	1e-51	-	-	DNA-binding transcriptional regulator, XRE-family HTH domain / 1.8	hp
76	45008	45223	+	hypothetical protein BN19_071 [Streptococcus phage SP-QS1]	3e-39	-	-	AAR2 C-terminal repeat region / 170	hp
77	45213	45404	+	hypothetical protein X878_0073 [Enterococcus phage VD13]	2e-36	-	-	Cdh1_DBD_1; Chromodomain helicase DNA-binding domain 1 / 14	hp
78	45467	45715	+	hypothetical protein FDH83_gp34 [Enterococcus phage IMEEF1]	6e-49	-	-	Bypass of Forespore C, N terminal /9.9	hp

79	45708	45974	+	hypothetical protein EnPhBC-611_gp11 [Enterococcus phage BC611]	6e-58	-	-	DarA_C ; Defence against restriction A C-terminal / 0.23	hp
80	45976	46281	+	DNA binding protein [Enterococcus phage IMEEF1]	4e-66	NTP-PPase_u3, Nucleoside Triphosphate Pyrophosphohydrolase, (5-79)	7.42e-28	Hypothetical protein ypjD; Pyrophosphatase, YPJD / 1.8e-10	Putative Pyrophosphatase
81	46378	46875	+	HNH endonuclease [Enterococcus phage vB_EfaS_IME198]	4e-32	HNH_3, HNH endonuclease, (57-99)	5.28e-08	HNH homing endonuclease; HNH catalytic motif, Helix-turn-helix DNA binding domain, protein-DNA complex / 5.8e-28	HNH endonuclease
82	47066	46932	-	-	-	-	-	Csm1_B; Csm1 subunit domain B / 72	hp
83	47125	47262	+	-	-	-	-	Sec61 protein translocation complex, beta subunit / 1.6	hp
84	47411	47527	+	-	-	-	-	Domain of unknown function (DUF4795) / 6.7	hp
85	47631	47951	+	hypothetical protein EnPhBC-611_gp09 [Enterococcus phage BC611]	4e-67	-	-	LydB; LydA-holin antagonis / 14	hp
86	47944	48048	+	hypothetical protein EnPhBC-611_gp60 [Enterococcus phage BC611]	4e-13	-	-	Ins_allergen_rp ; Insect allergen related repeat, nitrile-specifier detoxification / 25	hp
87	48051	48437	+	hypothetical protein FDH83_gp29 [Enterococcus phage IMEEF1]	5e-87	-	-	Designed dimeric coiled coil peptide with two terpyridine side chains; designed peptide, synthetic / 0.12	hp
88	48430	48801	+	hypothetical protein FDH83_gp28 [Enterococcus phage IMEEF1]	6e-77	-	-	Rossmann 2x3 fold protein; Structural Genomics / 31	hp

89	48879	49013	+	hypothetical protein FDH83_gp27 [Enterococcus phage IMEEF1]	9e-22	-	-	COG5346; Uncharacterized membrane protein [Function unknown] / 14	hp
90	49042	49140	+	-	-	-	-	LegK7; translocated effector, Ser/Thr protein kinase / 7.8	hp
91	49157	49309	+	hypothetical protein BN19_083 [Streptococcus phage SP-QS1]	3e-22	-	-	Beta propeller; bladed beta propeller, unknown function / 3.6	hp
92	49445	49879	+	hypothetical protein X878_0084 [Enterococcus phage VD13]	3e-77	-	-	Family of unknown function (DUF6120) / 140	hp
93	49866	50075	+	hypothetical protein BN19_085 [Streptococcus phage SP-QS1]	5e-41	-	-	ACRIIC5Nch; Anti-CRISPR, CRISPR-Cas, Cas9, inhibition / 0.94	hp
94	50113	50235	+	-	-	-	-	NqrC; Na+-transporting NADH:ubiquinone oxidoreductase, subunit NqrC / 13	hp
95	50331	50633	+	hypothetical protein AVT94_gp11 [Enterococcus phage vB_EfaS_IME198]	2e-58	-	-	Cell division protein FtsB; bacterial cell division, peptidoglycan synthesis, membrane protein complex, MEMBRANE PROTEIN /3.5	hp
96	50898	50990	+	-	-	-	-	DUF3660; Receptor serine/threonine kinase / 17	hp

97	51115	50987	-	hypothetical protein FDG77_gp88 [Enterococcus phage VD13]	2e-09	-	-	G0-G1_switch_2 ; G0/G1 switch protein 2 / 40	hp
98	51279	51112	-	-	-	-	-	ND3; Complex-I, ELECTRON TRANSPORT; HET: PTY, CDL, PC7, 8Q1; 2.97A {Polytomella sp. Pringsheim 198.80} / 120	hp
99	51615	51394	-	hypothetical protein FDH85_gp11 [Enterococcus phage SAP6]	2e-09	-	-	HTH-type transcriptional repressor phnF; PhnF, GntR, HutC, transcription, regulator, UTRA, DNA-bindin / 96	hp
100	51928	51749	-	hypothetical protein X878_0087 [Enterococcus phage VD13]	1e-32	-	-	LsbB; Bacteriocin, antimicrobial peptide, receptor binding domain / 120	hp
101	52567	52076	-	NUMOD4 motif family protein [Enterococcus phage VD13]	2e-49	HNH_3, HNH endonuclease, (54-98)	2.85e-06	HNH homing endonuclease; HNH catalytic motif, Helix-turn-helix DNA binding domain / 40e-30	hp
102	52852	52640	-	hypothetical protein X878_0088 [Enterococcus phage VD13]	4e-37	-	-	Protein of unknown function (DUF3087) / 17	hp
103	53030	52854	-	-	-	-	-	Putative Rab5-interacting protein / 4	hp
104	53898	53077	-	hypothetical protein X878_0090 [Enterococcus phage VD13]	3e-159	Smc, Chromosome segregation ATPase, (4-167)	3.75e-06	Predicted coiled-coil protein [Function unknown] / 0.27	hp

105	54731	53973	-	HNH endonuclease [Bacillus mojavensis]	6e-21	HNH endonuclease, (137-180)	2.07e-04	HNH homing endonuclease n=1 Tax=Enterococcus phage EF-P29 TaxID=1932891 RepID=A0A1L7DQ76_9CAUD / 3/2e-142	HNH homing endonuclease
106	55008	55136	+	-	-	-	-	Uncharacterized protein n=2 Tax=unclassified Saphexavirus / 4.6e-34	hp
107	55487	55182	-	-	-	-	-	Uncharacterized protein n=1 Tax=Enterococcus phage vB_EfaS_Ef2.2 / 7.5e-64	hp
108	55855	55484	-	ABC transporter [Enterococcus phage VD13]	1e-18	-	-	Uncharacterized protein n=1 Tax=Enterococcus phage vB_EfaS_IME198 / 3.4e-45	Hp
109	56115	55855	-	thioredoxin domain [Enterococcus phage VD13]	1e-53	NrdH-redoxin (NrdH) family; (3-79)	2.37e-17	GLUTAREDOXIN-LIKE PROTEIN NRDH; ELECTRON TRANSPORT, NRDH, THIOREDOXIN / 2.7e-10	thioredoxin domain
110	56277	56128	-	hypothetical protein X878_0006 [Enterococcus phage VD13]	4e-21	-	-	HalOD2; Halobacterial output domain 2 / 0.43	hp
111	56661	56281	-	methyltransferase [Enterococcus phage SAP6]	3e-49	-	-	6Z26_A Spindle assembly abnormal protein 6 homolog; centriole, centrosome, cartwheel, coiled coil, complex, alpha helical, / 0.0000011 4ZXQ_B Tail needle protein gp26; Viral protein, P22, Tail Needle, Membrane penetration; 2.75A {Enterobacteria phage P22} / 0.0000042	hp

								6GAP_C Outer capsid protein sigma-1; cell attachment protein, reovirus sigma1, coiled coil, beta-spiral repeat, VIRAL PROTEIN / 98.65	
112	57053	56658	-	hypothetical protein AVT94_gp21 [Enterococcus phage vB_EfaS_IME198]	7e-58	-	-	Protein of unknown function (DUF1642) / 2e-09	hp
113	57310	57053	-	hypothetical protein X878_0009 [Enterococcus phage VD13]	1e-48	-	-	Putative ESAT-6-like protein 7; Structural Genomics / 4.4	hp
114	57624	57310	-	hypothetical protein BN19_105 [Streptococcus phage SP-QS1]	3e-62	-	-	LXG domain of WXG superfamily / 3.5	hp
115	57664	57762	+	-	-	-	-	Protein of unknown function (DUF565) / 25	hp

Supplementary table S2. Oligonucleotide primers used for cloning the PlySE, PlyIME-SE and PlySheep-SE endolysin genes.

gene	vector	Oligonucleotide primer name	Oligonucleotide primer sequence (5'-3')
plySE	pBAD18	Fd plySE	GCTCGGTACCCGGGGATCCTAAGGAGATATACATATGAATCACAAAGTGCATCATCATCATCATCATATCGAAGGTAGGGGTACCATGGTTAAAGTAAACGATGTAG
		Rv plySE	CTCATCCGCCAAAACAGCCATTATAACTTAACTTGTGGGTAAG
plyIME-EF1-CHAP_lysSE-CBD	pBAD18-PlySE	Fd plyIME-EF1	CATATCGAAGGTAGGGGTACCATGGTTAAATTAAATGATGTACTAAGCTACGTGAATGGT
		Rv plyIME-EF1_EAD	GCAAGTGGTTTAGGCGTTGATGCGGCTTCGTACGGTGG
		Fd linker_CBD_plySE	TCAACGCCTAAACCACTT
plyShip-CHAP_lysSE-CBD	pBAD18-PlySE	Fd plyShip	CATATCGAAGGTAGGGGTACCATGGTCAAAAAGTCAGATGTTG
		Rv plyShip_EAD	GCAAGTGGTTTAGGCGTTGACTCGAGCGCCTCATAATT
